# MicroRNA-29b Regulates the Expression Level of Human Progranulin, a Secreted Glycoprotein Implicated in Frontotemporal Dementia

**DOI:** 10.1371/journal.pone.0010551

**Published:** 2010-05-10

**Authors:** Jian Jiao, Lauren D. Herl, Robert V. Farese, Fen-Biao Gao

**Affiliations:** 1 Gladstone Institute of Neurological Disease and Department of Neurology, University of California San Francisco, San Francisco, California, United States of America; 2 Gladstone Institute of Cardiovascular Disease and Departments of Medicine and of Biochemistry and Biophysics, University of California San Francisco, San Francisco, California, United States of America; 3 Biomedical Sciences Graduate Program, University of California San Francisco, San Francisco, California, United States of America; 4 Departments of Neurology and Neurobiology, University of Massachusetts Medical School, Worcester, Massachusetts, United States of America; National Institutes of Health, United States of America

## Abstract

Progranulin deficiency is thought to cause some forms of frontotemporal dementia (FTD), a major early-onset age-dependent neurodegenerative disease. How progranulin (PGRN) expression is regulated is largely unknown. We identified an evolutionarily conserved binding site for microRNA-29b (miR-29b) in the 3′ untranslated region (3′UTR) of the human *PGRN* (hPGRN) mRNA. miR-29b downregulates the expression of luciferase through hPGRN or mouse *PGRN* (mPGRN) 3′UTRs, and the regulation was abolished by mutations in the miR-29b binding site. To examine the direct effect of manipulating endogenous miR-29b on hPGRN expression, we established a stable NIH3T3 cell line that expresses hPGRN under the control of the cytomegalovirus promoter. Ectopic expression of miR-29b decreased hPGRN expression at the both mRNA and protein levels. Conversely, knockdown of endogenous miR-29b with locked nucleic acid increased the production and secretion of hPGRN in NIH3T3 cells. Endogenous hPGRN in HEK 293 cells was also regulated by miR-29b. These findings identify miR-29b as a novel posttranscriptional regulator of PGRN expression, raising the possibility that miR-29b or other miRNAs might be targeted therapeutically to increase hPGRN levels in some FTD patients.

## Introduction

Frontotemporal dementia (FTD) is associated with focal atrophy in the frontal and/or temporal lobes that alters personality and social behaviors and impairs language and cognition, among other clinical manifestations [1]. FTD is now recognized as the most common cause of early onset dementia in people under the age of 60, but effective treatment is still unavailable [2]. In addition to pathogenic mutations in *TAU* [3], [4], several disease genes have been identified that offer great hope for elucidating the pathogenic mechanisms of FTD and developing therapeutic approaches. These genes encode the AAA-type ATPase valosin-containing protein [5], the endosomal sorting complex required for transport III (ESCRT-III) component CHMP2B [6], and a secreted glycoprotein called progranulin [7], [8]. In addition, two RNA-binding proteins, TDP-43 [9], [10] and FUS [11], [12], have been identified in subsets of FTD brains as major ubiquitylated proteins in tau-negative cytoplasmic inclusions.

Among these pathogenic factors, progranulin stands out with two unique features. First, encoded by the *GRN* gene, it is the only secreted protein [13]. Second, the underlying mechanism of FTD associated with progranulin mutations is thought to be haploinsufficiency [7], [8], whereas other disease proteins seem to contribute to pathogenesis, at least in part, through toxic gain-of-function mechanisms [14]–[17]. Thus, molecular interventions that increase the production and secretion of progranulin from the remaining wildtype *GRN* allele are potential therapeutic strategies. In principle, progranulin expression and secretion in neurons or other cell types in the brain could be regulated by multiple mechanisms—none of which have been extensively investigated.

MicroRNAs (miRNAs) are small, noncoding RNAs that regulate gene expression mostly by binding to the 3′ untranslated region (3′UTR) of target mRNAs [18], [19]. Typically 21–23 nucleotides long, miRNAs repress translation or decrease the stability of their target mRNAs [18]–[20]. Some miRNAs evidently upregulate the expression of their target mRNAs by binding to 5′UTRs or coding regions [21], [22]. miRNAs are involved in gene expression in a wide range of cellular and developmental contexts, including the nervous system [23]–[25]. Although the human genome has 500 or more miRNAs, each predicted to target hundreds of mRNAs [18]–[20], few miRNA–target interactions have been validated experimentally. Because of their small size, miRNAs and their target sites are becoming attractive candidates for developing potential therapeutic approaches for human diseases [26].

In this study, we identified a miR-29b binding site in the 3′UTR of hPGRN mRNA. Both overexpression and locked nucleic acid (LNA) knockdown experiments demonstrated a role for miR-29b in regulating progranulin expression through its 3′UTR. Increased progranulin translation led to increased secretion. Thus, miR-29b is novel regulator of progranulin expression, raising the possibility of manipulating the activities of miR-29b and other miRNAs in the adult brain to treat FTD associated with progranulin deficiency.

## Results

### The 3′UTR of hPGRN mRNA Contains a Predicted miR-29b Binding Site

**Figure 1:**
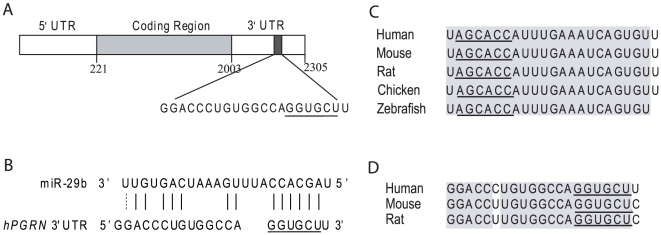
Identification of a miR-29b target site in the 3′UTR of hPGRN mRNA. (**A**) Schematic representation of hPGRN mRNA (NM_002087.2 showing with the predicted miR-29b binding site in the 3′UTR. (**B**) The actual nucleotide sequences in hPGRN mRNA and miR-29b show partial match. (**C**) Sequence alignment indicates that miR-29b is 100% conserved in vertebrates. (**D**) The predicted miR-29b binding site in hPGRN 3′UTR is highly conserved in mammals. The miR-29b seed sequences and their predicted binding sites in the hPGRN 3′UTR are shown underlined. The conserved nucleotides are in grey.

It remains challenging to use computational approaches to systematically and accurately predict the genome-wide targets of each miRNA and potential miRNAs that regulate a specific mRNA under physiological conditions [27]. To identify miRNAs that regulate hPGRN expression, we used an miRNA target prediction program that considers complementarity, target site accessibility, and the extent of evolutionary conservation [28]. This program predicted a putative binding site for miR-29b in the hPGRN 3′UTR, which contains about 300 nucleotides (Fig. 1A, B). MiR-29b is highly conserved in vertebrates, and its nucleotide sequence is 100% identical among several species (Fig. 1C). Although 3′UTR sequences tend to drift more rapidly during evolution [27], the putative binding sites for miR-29b in *PGRN* 3′UTRs are also highly conserved in mammals, with only one nucleotide difference between humans and rodents (Fig. 1D), suggesting an evolutionarily conserved miRNA–mRNA interaction with potentially important regulatory functions. Moreover, miR-29b is highly expressed in adult brains and in postmitotic neurons [29]. This expression pattern overlaps with that of progranulin [30]. Thus, miR-29b is a good candidate miRNA that may directly regulate progranulin expression. The following studies were intended to address this question in detail.

### miR-29b Suppresses Expression of the Luciferase Reporter Containing the hPGRN 3′UTR

**Figure 2:**
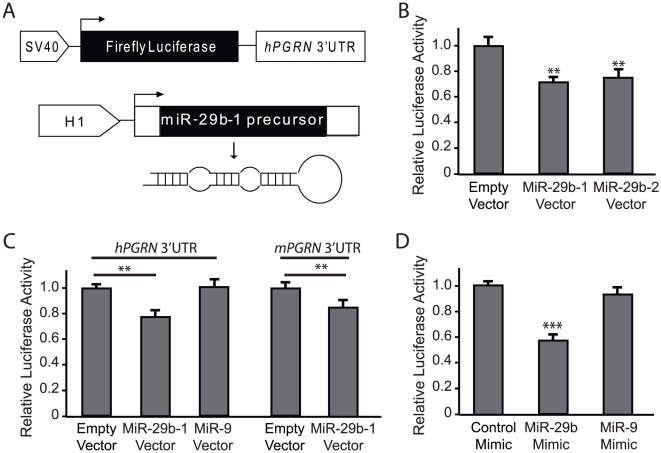
MiR-29b suppresses the expression of the luciferase reporter with hPGRN 3′UTR. (**A**) Schematic representation of the constructs used in the luciferase reporter assay. hPGRN 3′UTR was cloned into the luciferase vector containing the SV40 promoter, which was cotransfected with the vector encoding pre-miR-29b-1 and H1 promoter. (**B**) Both pre-miR-29b-1 and pre-miR-29b-2, two genes located on different chromosomes that produce the identical mature miR-29b, had similar effects on luciferase expression. (**C**) Coexpression of miR-29b but not miR-9 suppressed the expression of luciferase with hPGRN 3′UTR. miR-29b also suppressed the expression of luciferase with mPGRN 3′UTR. (**D**) Luciferase activity was also reduced by a miR-29b mimic but not by a miR-9 mimic. Cel-miR-67 (Dharmacon), which doesn't exist in mammals, was used as the control. Values are mean ± SEM. **P<0.01. ***P<0.001.

To validate the interaction between miR-29b and the hPGRN 3′UTR, we cloned hPGRN 3′UTR into the reporter vector to serve as the 3′UTR of the luciferase coding region (Fig. 2A). We also cloned the 421-nucleotide genomic fragment that contains pre-miR-29b-1 into the pSuper vector (Fig. 2A). Cotransfection of these two vectors into HEK293 cells decreased luciferase expression to a greater extent than a control vector that lacked miRNAs (Fig. 2B). Cotransfection of miR-29b-1 and the luciferase vector without hPGRN 3′UTR did not affect luciferase expression (data not shown). Thus, miR-29b-1 acts through hPGRN 3′UTR to regulate luciferase expression.

Mature miR-29b can be produced from two precursors encoded by two genes located on chromosomes 7 and 1, respectively. Expression of pre-miR-29b-1 or pre-miR-29b-2 suppressed the expression of luciferase with hPGRN 3′UTR (Fig. 2B), suggesting that both genes indeed can produce functional mature miR-29b to regulate hPGRN 3′UTR. No suppression was seen after cotransfection with a negative control vector expressing miR-9 [31], which has no predicted binding site in hPGRN mRNA (Fig. 2C). Since hPGRN and mPGRN mRNAs contain conserved binding sites for miR-29b (Fig. 1D), and the effects of miRNAs depend on the secondary structures of surrounding mRNA sequences, we also examined whether miR-29b could also interact with mPGRN mRNA. We cloned mPGRN 3′UTR into the luciferase reporter construct and found that indeed miR-29b also suppressed luciferase expression through mPGRN 3′UTR (Fig. 2C).

In addition to vector-based expression of pre-miR-29b-1, which produces mature miR-29b after being transfected into HEK293 cells, we also used miRNA mimics, which are double-stranded RNA oligonucleotides that are chemically modified with Dharmacon ON-TARGET (Themo Scientific Dharmacon). Transfection of miR-29b but not miR-9 mimics decreased luciferase reporter expression (Fig. 2D). For this experiment, mimics for cel-miR-67, which does exist in mammals, were used as the control. These findings raise the possibility that miR-29b may specifically interact with *hPGRN* 3′UTR.

### miR-29b Directly Interacts with the Predicted Binding Site in the hPGRN 3′UTR

**Figure 3:**
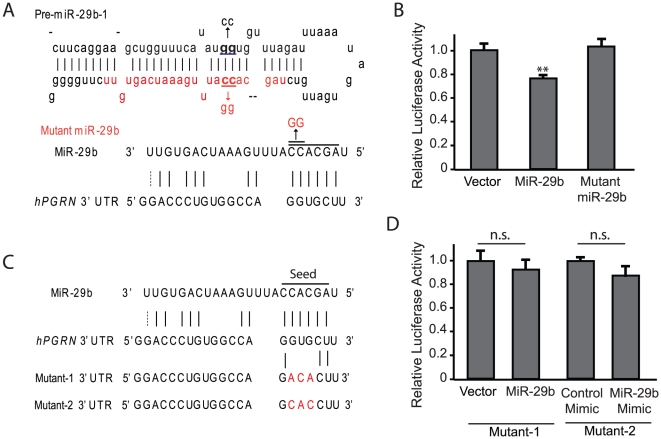
miR-29b directly targets hPGRN 3′UTR through the seed region. (**A**) The seed region of miR-29b and the opposite strand in the stem-loop in the precursor were mutated. (**B**) HEK293FT cells were transfected with luciferase constructs and wildtype or mutant miR-29b precursors. Luciferase activity was reduced by miR-29b but not by mutant miR-29b. (**C**) The seed region binding site in the hPGRN 3′UTR was mutated. Two different mutant 3′UTR were generated. (**D**) Mutant hPGRN 3′UTR did not respond to miR-29b suppression. Mutant 1 was used for experiment with the miR-29b vector. Mutant 2 was used for experiment with the miR-29b mimic. The seed region is underlined. **P<0.01. n.s. not significant.

To examine whether the interaction between miR-29b and hPGRN 3′UTR is direct or indirect, we generated mutations in miR-29b. In pre-miR-29b-1, we mutated two nucleotides in the miR-29b seed region from CC to GG (Fig. 3A). To ensure that mutant pre-miR-29b-1 maintains its stem loop structure so that it can be properly processed to produce mature miR-29b, we also mutated GG in the opposite strand of the stem into CC (Fig. 3A). Cotransfection of normal but not the mutant pre-miR-29b-1 suppressed luciferase reporter expression (Fig. 3B). Moreover, we also mutated the miR-29b binding site in hPGRN 3′UTR in which GTG was changed to ACA (Fig. 3C). With such mutations present in hPGRN 3′UTR, luciferase expression failed to be regulated by miR-29b produced from the vector expressing its precursor (Fig. 3D). Moreover, miR-29b mimics were unable to suppress luciferase expression with a different mutant hPGRN 3′UTR in which GTG in the miR-29b binding site was mutated to CAC (Fig. 3C, D). These experiments demonstrate that miR-29b interacts directly with the binding site in hPGRN 3′UTR to regulate luciferase reporter expression.

### miR-29b Suppresses the Production and Secretion of hPGRN

Next, we examined the regulation of endogenous hPGRN by miR-29b. First, to more specifically investigate the regulation of hPGRN expression at the posttranscriptional level, we cloned hPGRN with its full 3′UTR into the pcDNA3.1(−) vector, in which hPGRN is under the control of the pCMV promoter. We transfected this plasmid into NIH3T3 cells and established stable cell lines (hPGRN-3T3) in which constitutive hPGRN expression was relatively constant, enabling greater sensitivity for detecting miRNA-induced changes in expression at the post transcriptional level.

**Figure 4:**
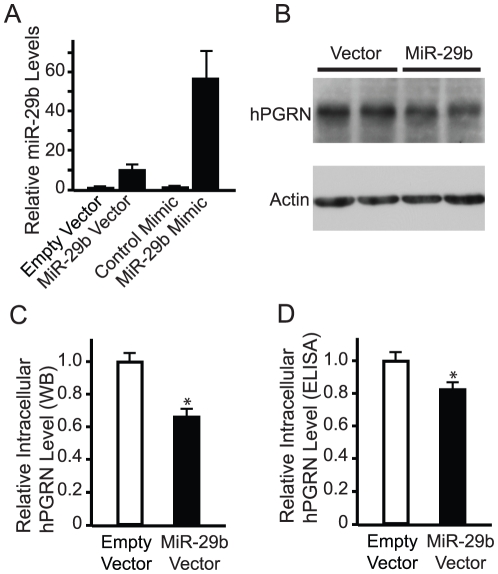
MiR-29b decreases the levels of intracellular and secreted hPGRN. (**A**) The relative levels of mature miR-29b in hPGRN-3T3 cells were increased by transient expression of the miR-29b precursor or miR-29b mimic. (**B**) Western blot analysis showing that the lower intracellular levels of hPGRN in hPGRN-3T3 cells after ectopic expression of pre-miR-29b-1. Actin was used as the loading control. (**C**) Quantification of relative intracellular hPGRN levels with or without the overexpression of pre-miR-29b-1. n = 4. WB: western blot. (**D**) The relative intracellular levels of hPGRN with or without overexpression of pre-miR-29b-1 were also measured by ELISA.

Transfection of hPGRN-3T3 cells with the pre-miR-29b-1 vector significantly increased the level of mature miR-29b (Fig. 4A). Correspondingly, the level of intracellular hPGRN as detected on western blot was decreased by about 35% (Fig. 4B, C). Although NIH3T3 cells also express PGRN, the antibody we used recognized transfected hPGRN but not its mouse counterpart expressed endogenously in NIH 3T3 Cells. We repeated the transfection experiments and measured hPGRN levels with ELISAs. Again, miR-29b significantly reduced the level of intracellular hPGRN in hPGRN-3T3 cells (Fig. 4D).

**Figure 5:**
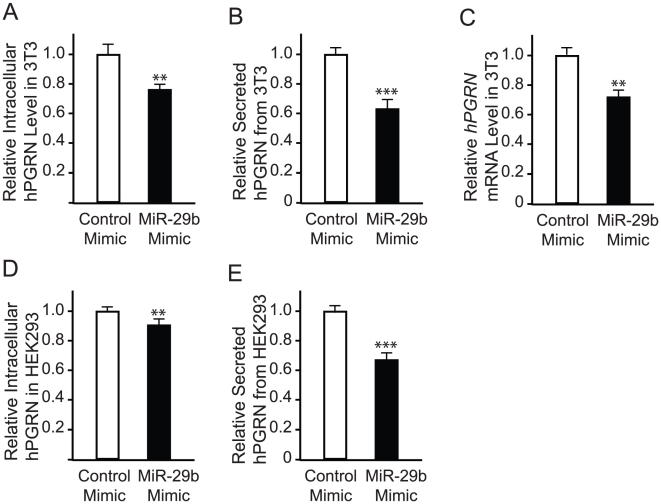
(**A**, **B**) hPGRN-3T3 cells were transfected with miR-29b or negative control mimics, and the relative hPGRN levels in total cell lysates (**A**) and medium (**B**) were determined by ELISA. In all cases, the relative level of the control was set as 1.0. Values are mean ± SEM. *P<0.05. **P<0.01. ***P<0.001. (**C**) Quantitative RT-PCR analysis revealed that the level of hPGRN mRNA was decreased by miR-29b mimics. (**D, E**) The relative hPGRN levels in total cell lysates (**D**) and medium (**E**) of HEK293 cells as determined by ELISA after transfection with miR-29b or control mimics. Value are mean ± SEM. ** *P*<0.01, *** *P*<0.001. n = 12.

We also used miR-29b mimics to increase the level of mature miR-29b (Fig. 4A). The levels of intracellular hPGRN measured by ELISA were significantly decreased by miR-29b mimics but not by control mimics (Fig. 5A). Since progranulin is a secreted molecule and its reduced trophic function is thought to underlie the pathogenesis of FTD [7], [8], we wondered whether miRNA regulation of hPGRN expression also affects the level of its secreted form. As expected, an increased level of miR-29b led to a lower level of hPGRN in the medium (Fig. 5B), correlating with the decreased expression in hPGRN-3T3 cells. miRNAs can regulate gene expression through either translational suppression or mRNA stability or both. The decrease in the level hPGRN protein as we observed is likely due to a decrease in hPGRN mRNA stability since we found by quantitative RT-PCR that the level of hPGRN mRNA was also decreased by miR-29b mimics (Figure 5C).

To examine whether miR-29b also regulates the expression of endogenous hPGRN, we transfected the human cell line HEK293 cells with miR-29b mimics and measured the levels of intracellular and secreted hPGRN. To ensure that we measured the changes in hPGRN levels after the treatment with miR-29b mimics, we changed culture medium 24 h after transfection. During our studies, we noticed that the transcriptional regulation of endogenous hPGRN expression in HEK293 and other cell lines is sensitive to extracellular stimuli and different experimental manipulations (J. Jiao, unpublished observations), thus, we performed this experiment with a high sample number (n = 12 per treatment). Indeed, we found that indeed the expression levels of endogenous intracellular hPGRN (Fig. 5D) and secreted hPGRN (Fig. 5E) were decreased by miR-29b mimics.

### miR-29b Knockdown Increases the Levels of Intracellular and Secreted hPGRN

**Figure 6:**
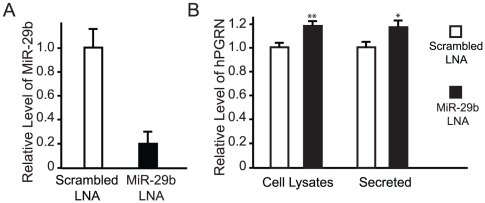
Knockdown of endogenous miR-29b led to increased production and secretion of hPGRN. (**A**) qRT-PCR confirmed that mature miR-29b level was decreased by about 80% in hPGRN-3T3 cells transfected with miR-29b-specific LNA. (**B**) hPGRN-3T3 cells were transfected with miR-29b-specific or scrambled LNA knockdown probes, and the hPGRN levels in total cell lysates and medium were determined by ELISA. Values are mean ± SEM. *P<0.05, **P<0.01. n = 4.

Since ectopic expression of miR-29b suppressed hPGRN expression, we used locked nucleic acid (LNA)-mediated miRNA silencing to determine whether loss of endogenous miR-29b activity enhances hPGRN expression in stably transfected hPGRN-3T3 cells. Transfection of miR-29b-specific LNA probes reduced endogenous miR-29b levels by about 80% (Fig. 6A). Correspondingly, the intracellular level of hPGRN increased by about 19%, as measured by ELISA (Fig. 6B). The level of secreted hPGRN increased to a similar extent after miR-29b knockdown (Fig. 6B). Thus, hPGRN expression can be regulated by manipulating the activity of endogenous miR-29b.

## Discussion

About 5–10% of familial cases of FTD are caused by mutations in *GRN* [2]. Many pathogenic mutations reduce hPGRN levels, indicating a haploinsufficiency pathogenic mechanism [7], [8]. In carriers of *GRN* mutations, the normal allele of *GRN* is still functional. Thus, molecular interventions that increase the production and secretion of hPGRN from the normal gene are conceivably potential therapeutic avenues. However, very little is known about how hPGRN expression is regulated at the transcriptional and translational levels. Here we provide multiple lines of experimental evidence to demonstrate that miR-29b is a novel regulator of hPGRN production.

We identified miR-29b with a miRNA target prediction program that takes into account the secondary structures of target mRNAs and seed region complementarity [28]. We also confirmed that miR-29b interacts directly with the hPGRN 3′UTR and regulates the expression level of endogenous hPGRN. An important experimental approach in this study is the use of a stable NIH3T3 cell line that expresses hPGRN under the control of the CMV promoter. During our study, we noticed that the level of endogenous progranulin is very sensitive to many extracellular stimuli, which makes it difficult to examine the specific effects of miRNAs. The use of the stably transfected cell line allowed us to bypass the effects of our experimental manipulation on the transcription of endogenous progranulin and focus on the effects of miRNAs on posttranscriptional regulation of progranulin expression with a more sensitive system. As expected, the effect of miR-29b on progranulin expression is not as dramatic as that of transcription factors, consistent with the notion that in many cases, miRNAs fine tune gene expression [20], [27].

MiR-29b is downregulated in several types of tumor cells [32]–[34], which is in reverse correlation with the increased progranulin expression in tumor cells [35]. Thus, the regulatory interaction between miR-29b and hPGRN may exist during tumorigenesis as well. In the nervous system, miR-29b is developmentally regulated, with the highest level in adult mouse brain [29]. Interestingly, the miR-29a/b-1 cluster is significantly decreased in the brains of Alzheimer's disease patients, and the decrease correlates with the increased level of β-secretase [29]. MiR-29b regulates several other mRNA targets as well [33], [34], [36], [37], consistent with the notion that each miRNA targets many mRNAs in different cellular and developmental settings [27]. Thus, caution is needed when considering miRNAs as potential therapeutic target. In the case of progranulin, the potential effects of miR-29b knockdown on other mRNA targets and biological processes must be considered. With that stated, miRNAs are attractive potential drug targets for FTD because antigomirs are relative small and have been used successfully in in vivo models of cardiovascular diseases [38].

Two other miRNAs, miR-588 and miR-615-5p, were predicted to target hPGRN mRNA by more than two target prediction programs. miR-588 but not miR-615-5p also suppressed hPGRN expression in stable cell lines (data not shown), indicating that multiple miRNAs may be potential targets for therapeutic manipulation of hPGRN levels. Indeed, miR-659 also inhibits hPGRN expression [39]. Interestingly, a common genetic variant (rs5848) in the binding site of miR-659 increases the inhibition of hPGRN expression by miR-659 [39]. Thus, alterations in the miRNA pathway may contribute to the molecular pathogenesis of FTD, although the specific role of this genetic variant (rs5848) in the pathogenesis of FTD remains to be clarified [39], [40].

It seems certain that, in addition to miRNA regulation, the production and secretion of hPGRN is regulated at multiple levels by several other mechanisms. It will be important to investigate all these molecular pathways to identify the most promising molecular targets to increase the levels of hPGRN in patients with FTD.

## Materials and Methods

### Cell Culture

HEK293FT cells (Invitrogen) were maintained in Dulbecco's modified Eagle medium (Gibco) supplemented with 10% fetal bovine serum. An hPGRN-expressing NIH-3T3 stable cell line (hPGRN-3T3) was established by transfecting pcDNA3.1(−) containing full-length hPGRN cDNA (including the 3′UTR) into NIH3T3 cells (Invitrogen) and selecting cells with G418 for 3 weeks.

### DNA Constructs

The full-length 3′UTRs of hPGRN and mPGRN mRNAs were amplified from cDNAs of HEK293FT or genomic DNAs from mouse ES cells by PCR. 5′-TCTAGAGGGACAGTACTGA AGACT-3′ (forward primer) and 5′-TCTAGAGAAAGTGTACAAACTTT ATTG-3′ (reverse primer) were used to amplify hPGRN 3′UTR and 5′-TCTAGA GGAAGGGCTACAGACTTA-3′ (forward primer) and 5′-TCTAGAGAAAGTGTA CAAACTTTATTG-3′ (reverse primer) were used to amplify mPGRN 3′UTR. The primers were designed to put an XbaI restriction enzyme site at both the 5′ and 3′ ends of the PCR products, which were then subcloned into the XbaI site of the PGL3 promoter vector containing the luciferase reporter (Promega).

MiR-29b precursors were amplified from HEK293FT genomic DNA using 5′-GTCGA CCTGACTGCCATTTG-3′ and 5′-ATCGA TGCTCTCCCATCAATA-3′ for pre-miR-29b-1 on chromosome 7 and 5′-GTCGACT GTGTTTATTTTAAACACAA-3′ and 5′-ATCGATTGAATCTCCCTTCT TTCTT-3′ for pre-miR-29b-2 on chromosome 1. SalI and ClaI restriction enzyme sites were placed at the ends of the PCR products for subcloning into the pSuper basic vector (OligoEngine).

Full-length hPGRN cDNA with the 3′UTR in pCMV-SPORT6 was from Open Biosystems (Thermo Scientific). The hPGRN cDNA was cloned into the pcDNA3.1(−) vector between the EcoRI and HindIII sites.

### Mutagenesis

Mutagenesis of miR-29b and its putative target site in hPGRN 3′UTR was carried out with the QuickChange Multi Site-Directed Mutagenesis kit (Stratagene) according to the manufacturer's instructions. For mutagenesis of miR-29b, 5′-CCCAAGA ACACTGATTTCAAATCCTGCTAGAC AATCAC-3′ and 5′-AATCTA AACCAGG ATATGAAACCAGCTTCCTGAAGAA GC-3′ were used. For mutagenesis of the miR-29b target site in hPGRN 3′UTR, 5′-GACCCTGTGGCCAGACACTTTTCC CTATCCACAG-3′ was used.

### Dual Luciferase Assay

HEK293FT cells were placed in 24-well plates the day before transfection. Firefly luciferase expression vectors (PGL3; 200 ng), miR-29b-pSuper or pSuper empty vector (200 ng), and Renilla luciferase expression vector (50 ng) were cotransfected into the cells with Lipofectamine 2000 (Invitrogen) following the manufacturer's instructions. Luciferase assays were performed 24 or 48 h later with the Dual Luciferase Reporter Assay System (Promega) as directed by the manufacturer. Firefly luciferase activity was normalized to Renilla luciferase activity. The experiments were carried out in quadruplicate.

### Quantitative RT-PCR

Total RNAs were isolated from the hPGRN-3T3 stable cell line using the miRNeasy mini kit (Qiagen) according to the manufacturer's instruction. Total RNAs (10 ng) was reverse transcribed into cDNA with the TaqMan microRNA reverse transcription kit (Applied Biosystems) with miR-29b, miR-126 or U6 specific RT primers. The cDNAs were then used for real-time PCR reaction with Taqman MicroRNA assay kits specific for miR-29b, miR-126, and U6 (Applied Biosystems). miR-29b levels were normalized to miR-126 or U6 levels.

To quantify hPGRN mRNA levels, total RNAs were isolated from the hPGRN-3T3 stable cell line using the RNeasy mini kit (Qiagen) according to the manufacturer's instruction. Total RNAs (1 ug) were reverse transcribed into cDNA with the TaqMan reverse transcription kit (Applied Biosystems) using random hexamers primers. The cDNAs were then used for real-time PCR reaction with SYBR green PCR master mix (Applied Biosystems) using hPGRN primers 5′- CCTGGACCCCGGAGGAGC-3′ and 5′-ACGGTAAAGATGCAGGAGTGG-3′, and human GAPDH primers 5′- TGCACCACCACCTGCTTAGC-3′ and 5′- GGCATGGACTGTGGTCATGAG-3′. hPGRN levels were normalized to hGAPDH levels.

### Western Blotting

Total cell lysates were prepared using RIPA buffer (50 mM Tris, pH 7.5, 150 mM NaCl, 1% NP-40, 0.5% sodium deoxycholate, 0.1% sodium dodecyl sulfate) with protease inhibitor cocktail (Thermo Scientific). Protein samples were separated by SDS-PAGE with 10% gels and transferred to polyvinylidine fluoride (PVDF) membranes. The following antibodies were used: rabbit polyclonal anti-hPGRN (1∶1000; Alexis Biochemicals); goat polyclonal anti-hPGRN (1∶1000; R&D Systems) and mouse monoclonal anti-actin (1∶5000; Sigma).

### Enzyme-linked Immunosorbent Assay (ELISA)

hPGRN-3T3 stable cells (1.2×10^5^ cells per well) were seeded in 12-well plates. The next day, the medium was changed to fresh Dulbecco's modified Eagle medium with 10% fetal bovine serum, and the cells were transfected 2 h later with 100 pmol of miRNA mimics (Dharmacon) (final concentration in the medium was 100 nM) or miRCURY LNA microRNA knockdown probes (Exiqon) (final concentration in the medium was 100 nM), using Lipofectamine 2000 (Invitrogen) as directed by the manufacturer. Total cell lysates were prepared with RIPA buffer 48–72 h later, and the medium was centrifuged at 13,000 rpm for 1 min to remove cell debris. Total cell lysates and culture medium were diluted 1∶50 and 1∶100, and hPGRN concentrations were detected with an ELISA kit (Alexis Biochemicals) according to the manufacturer's instructions.

### Measurement of endogenous hPGRN levels affected by miR-29b

HEK293FT cells (1.2×10^5^ cells per well) were seeded in 12- well plates. The next day, the medium was changed to fresh Dulbecco's modified Eagle medium (DMEM) with 10% fetal bovine serum (FBS), and the cells were transfected 2 h later with 100 pmol of control or miR-29b miRNA mimics (Dharmacon) using Lipofectamine 2000 (Invitrogen) as directed by the manufacturer. The medium was changed to fresh DMEM with 10% FBS 24 h after the transfection, and the medium and cell lysates were collected 6 h later. Total cell lysates were prepared with RIPA buffer, and the medium was centrifuged at 13,000 rpm for 1 min to remove cell debris. Total cell lysates and culture medium were diluted 1∶50, and hPGRN concentrations were detected with an ELISA kit (Alexis Biochemicals) according to the manufacturer's instructions.
